# Quantifying Color Vision Changes Associated With Cataracts Using Cone Contrast Thresholds

**DOI:** 10.1167/tvst.9.12.11

**Published:** 2020-11-03

**Authors:** Urmi Mehta, Anna Diep, Kevin Nguyen, Bryan Le, Clara Yuh, Caroline Frambach, John Doan, Ang Wei, Anton M. Palma, Marjan Farid, Sumit Garg, Sanjay Kedhar, Matthew Wade, Kailey A. Marshall, Kimberly A. Jameson, M. Cristina Kenney, Andrew W. Browne

**Affiliations:** 1 Gavin Herbert Eye Institute, Department of Ophthalmology, University of California, Irvine, Irvine, California, USA; 2 Western University of Health Sciences, Pomona, California USA; 3 University of California, Irvine School of Medicine, Irvine, California USA; 4 Creighton University School of Medicine, Omaha, Nebraska, USA; 5 Drexel University College of Medicine, Philadelphia, Pennsylvania, USA; 6 Medical College of Wisconsin, Wauwatosa, Wisconsin, USA; 7 Institute for Clinical and Translational Sciences, University of California, Irvine, Irvine, California, USA; 8 Institute for Mathematical Behavioral Sciences, University of California, Irvine, Irvine, California, USA; 9 Department of Biomedical Engineering, University of California, Irvine, Irvine, California, USA

**Keywords:** cone contrast test, cone contrast threshold, color vision changes before and after cataract surgery, color vision changes associated with aging, age-related decline in color vision

## Abstract

**Purpose:**

To evaluate effects of age and simulated and real cataractous changes on color vision as measured by the high-definition cone contrast test (CCT).

**Methods:**

Twenty-four healthy volunteers from two cohort studies performed CCT using best-corrected visual acuity, filters, mydriasis, and pinhole correction. Retrospective cross-sectional study of patients seen in eye clinics evaluated the relationship between age and color vision, and age and lens status in 355 eyes. Last, 25 subjects underwent CCT before and after cataract surgery.

**Results:**

CCT scores were most reliable in the nonmydriatic condition without pinhole correction. Progressively dense brown filters produced small decreases in S-cone sensitivity. Linear regression analysis of phakic subjects showed a decline for all cone classes with age. Rate of decline was greater for S-cones (slope = −1.09; 95% confidence interval [CI], −1.30 to 0.86) than M-cones (slope = −0.80; 95% CI, −1.03 to −0.58) and L-cones (slope = −0.66; 95% CI, −0.88 to −0.44). CCT scores increased for S-cones but reduced for L- and M-cones in pseudophakic subjects compared with phakic patients. CCT scores after cataract surgery increased for S-cones, M-cones, and L-cones by 33.0 (95% CI, 8.6 to 57.4), 24.9 (95% CI, 3.8 to 46.0), and 22.0 (95% CI, −3.2 to 47.3), respectively.

**Conclusions:**

CCT assessment allows for clinically practical quantitation of color and contrast vision improvement after cataract surgery and aging patients who note poor vision despite good visual acuity.

**Translational Relevance:**

CCT testing, which quantifies hereditary and acquired color deficiency, can also quantify the degree of cataract severity and, combined with other parameters, can provide more precise guidance for cataract extraction to optimize patient care.

## Introduction

Color vision is an integral part of visual function that gradually decreases with age.^1^^–^^3^ However, visual fields and acuity are the only elements routinely measured or considered in clinical trials. Senescent changes, including senile miosis and increased density and opacity of optical media,^2^^,^^4^ diminish the amount of light reaching the retina where visual transduction initiates visual acuity, contrast sensitivity, and color vision.^5^ The most common etiology for decreased media clarity is lenticular opacification. With age, the translucent lens begins to yellow and harden toward brunescence, increasing its absorption of short-wavelength visible light, causing acquired tritan deficits.^6^^–^^8^ Increasing lens density and the eventual progression to cataracts increase forward light scattering, decreasing retinal illumination, contrast sensitivity, and visual acuity while increasing glare.^2^

Most studies of age-related changes in color vision were performed using investigational color vision assays that are not available routinely in clinics. The anomaloscope, considered the quantitative gold standard” for assessing color vision deficiency (CVD), is a matching test that quantifies abnormalities along the deutan and protan (red–green) axis.^9^ Extensive training to administer, high equipment cost, and long test duration yield anomaloscopy a rarely used tool outside research settings. Instead, pseudoisochromatic plates are used because they are cost effective and easy to administer. Although the Ishihara and most other pseudoisochromatic plates use multicolored dots along protan and deutan confusion lines, they do not readily specify type or severity of CVD. The Hardy–Rand–Rittler, however, offers some quantification of type and severity.

The Farnsworth–Munsell 100-Hue arrangement test is more discriminative than pseudoisochromatic plates. It requires subjects to arrange 85 different colored caps in four different groups by order of their hue.^10^ Recent adaptations to this test into software have improved previous setbacks, such as length of assessment time and wear and tear to physical pigmented cards, leading to hue confusion.^11^ However, performance complexity, long test duration, and subjectivity in determining the degree of CVD^9^ render it impractical in routine clinical practice. Furthermore, the Farnsworth–Munsell 100-Hue correlates significantly with nonverbal intelligence.^12^ Shorter versions of this test include the saturated Farnsworth D15 and desaturated Lanthony D15 tests.

Computerized programs, like the custom system used by Fristrom and Lundh,^2^ which displayed colored letters on a computer monitor, found a quantifiable negative impact on color sensitivity in subjects undergoing cataract surgery that was greatest for the tritan axis. These prior studies using custom computerized testing or traditional color vision tests are either not broadly available, clinically practical, or provide sufficient and reliable information.

The more recent introduction of the cone contrast threshold (CCT) test answered the challenges posed by traditional color vision tests by allowing for faster and easier testing while producing accurate quantitation and characterization of CVD.^13^ The original CCT presented a series of colored letters visible to a single cone type in graded steps of cone contrast. Charts for each cone type consisted of 10 rows of colored letters that incrementally diminished in contrast by 0.1 log steps per row.^14^^,^^15^ Scoring was predicated on threshold techniques utilizing the British Standards Institute (Bailey–Lovie) letters with equal legibility.^15^ The Rabin CCT (RCCT) was also based on a Cooperative Research and Development Agreement and technology available around 2010. It has been used to accurately detect both hereditary and acquired causes of CVD, such as aging,^17^ glaucoma,^18^ and age-related macular degeneration. The RCCT even has the capability of detecting early stages of ocular, systemic, and neurologic diseases^15^ through decreased cone contrast sensitivity. The CCT high definition (HD) is a practical implementation stemming from the findings originally published with the RCCT.

CCT testing differentiates itself from prior testing modalities because it rapidly yields continuous quantitative data for all three cone classes. Recent advances in precise computer-based testing and system affordability have yielded logistically practical and readily available clinical visual function test devices to quantify and report quantitative changes in color and contrast vision. This work presents findings from a systematic investigation of media opacity on color vision using a conventional and clinically practical CCT system, ColorDx CCT HD (Konan Medical, Irvine, CA). The CCT HD was a collaborative development under the Cooperative Research and Development Agreement with the US Air Force, School of Aerospace Medicine, Operational Based Vision Assessment Team at Wright Patterson Air Force Base, to provide increased reliability and resolution of color vision testing with an expanded low-contrast range not available with the original RCCT. Precision pilot assessment was the original focus of CCT HD. However, efficient and highly granular assessment of acquired deficiencies, even in normal color vision ranges, may have useful clinical implications for functional vision testing in disease and drug or substance toxicity conditions.^16^

This study sought to quantify the influence of age and cataractous media opacities on color and contrast vision. Color vision changes were first quantified in healthy subjects with simulated media opacities and in normal healthy patients from all age groups. A cohort of healthy patients across a wide age range was studied to evaluate color vision changes with age. Finally, color vision changes associated with cataract surgery were quantified before and after surgery.

## Methods

This study received Institutional Review Board approval from the University of California, Irvine, and was conducted in accordance with the Declaration of Helsinki. Studies performed were compliant with the Health Insurance Portability and Accountability Act of 1996 and all participants enrolled in the perioperative CCT testing provided informed written consent. Three separate analyses were performed to evaluate the effects of cataracts and age on color vision.

## Subjects and Protocol

### Optimal CCT Test Conditions and Test Validation (Neutral Density Filters [NDs])

CCT testing conditions were optimized to determine if mydriasis affected CCT results, and if pinhole correction could be used to account for mydriasis or presbyopia during CCT testing. NDs were evaluated to confirm reduction in CCT scores with increased filter density. Thirty-six phakic eyes of 18 subjects (10 female, 8 male) between the ages of 23 and 38 years (25.3 ± 3.3 years) were recruited for this study. Testing was performed in triplicate under the following conditions: best-corrected visual acuity (no filter), distance best-corrected visual acuity with mydriasis (dilated), ND 0.3 (2× decrease in luminance), ND 0.9 (8× decrease), ND 1.5 (32× decrease) (Bernell Corp), and pinholes. ND 0.3, ND 0.9, and ND 1.5 have optical densities of 03, 09, and 15, respectively. Testing using ND was performed in order of decreasing luminance. One eye was temporarily patched while the fellow eye underwent testing. The inclusion criteria were (1) age 18 years or older, (2) no history of ocular disease or procedures, and (3) best-corrected visual acuity of 20/20 or better in the study eye.

### Simulated Cataractous Media Opacity (Brown Filters)

Cataract-like media opacities were simulated using brown filter lenses with increasing optical density. The inclusion criteria were the same as described for CCT testing optimization. Six phakic eyes of six additional healthy subjects (five female, one male) between the ages of 23 and 29 years without any ocular history were recruited for this study. Right eyes were tested under the following conditions: no filter, filter 1, filter 2, filter 3, filter 4 (a combination of filter 2 and filter 3 in series), and “blue blocker” filter (BPI, Empire Optical, North Hollywood, CA). The left eye remained patched throughout testing.

### Filter Spectral Transmission

The spectral transmission for the different ND and brown filters was obtained using a compact spectrometer (CCS200/M, THORLABS, Newton, NJ). Indirect sunlight from clear skies was used to create a baseline spectrum without photobleaching the sensor. The spectra from each filter were collected while maintaining the same position and angle of the sensor. Relative transmission of each filter was calculated. The spectral emission of the CCT monitor for red, green, and blue screens were also measured.

### Age and Lens Status Associations with Color Vision

The relationship between age and color vision in phakic and pseudophakic subjects was retrospectively reviewed in healthy patients attending optometry and medical clinics. Healthy phakic (*n* = 256) eyes were identified from the electronic medical record. Inclusion criteria for phakic subjects included: (1) age between 6 and 90 years, (2) no history of cataract extraction or surgery in study eye, and (3) no history of ocular disease in study eye other than age-related cataract formation. A second analysis evaluated color vision in older patients. Forty-two phakic and 57 pseudophakic eyes were identified and evaluated with CCT. Inclusion criteria for the second older age analysis included: (1) age 50 years or older, (2) history of age-related cataract surgery in the study eye, and (3) no history of ocular disease aside from cataract in study eye. Testing in pseudophakic subjects was performed well after (>3 months) the perioperative period from cataract surgery.

### Effects of Cataract on Color Vision

Finally, the effect of cataract was studied by evaluating CCT performance before and after cataract surgery. Thirty eyes (11 right, 19 left) from 25 patients (12 female, 13 male) between the ages of 51 and 84 years were recruited for this prospective study. The inclusion criteria were (1) age older than 50 years, (2) no history of retinal or macular pathology affecting central vision, and (3) completion of a postoperative visit. Subjects were excluded if their visual acuity decreased by more than two lines on Snellen at their postoperative month 1 visit. Visual acuity and color vision were assessed before surgery and 1 to 2 months after cataract extraction with intraocular lens (IOL) insertion to minimize the occurrence or retinal or corneal edema that may have developed in the proximal perioperative period. At their postoperative visit, pseudophakic subjects with a monofocal IOL targeted for distance vision performed CCT with near add correction, whereas patients with IOL selection for near vision, multifocal lens or extended depth of field lenses wore no reading add. Information regarding IOL type and color was obtained for 30 eyes and is listed in Table.

**Table.** IOL Tint and Type (*n* = 30). EDOF = Extended Depth of Field

IOL Tint and Type	
IOL color	Number
Clear	28
Yellow	2
IOL type	
Monofocal	23
Multifocal	4
EDOF	3

### Functional Testing

#### Cone Contrast Threshold

The ColorDx CCT HD (Konan Medical, Irvine, CA) is an adaptive visual function testing device that selectively stimulates retinal L-cones, M-cones, and S-cones. A white Dell desktop all-in-one computer and a 21.5” LG LED backlit IPS, 60 Hz color-calibrated anti-glare monitor with a maximum resolution of 1920 × 1080 pixels were used. The size of each Landolt C projected onto the center of the screen has a Snellen equivalent of 20/317, with a viewing angle of 1.43°. A series of tumbling Landolt-C optotypes were presented, with the gap in the “C” facing one of four randomized directions (up, down, left, or right) against a neutral (grey) background (approximately 74/135 cd/m^2^). Subjects indicated the perceived direction of the gap by pressing a keypad with arrows, with gated visibility of 5 seconds per stimulus as a forced choice to the subject, whether discernable or not. The optotype contrast, as LogCS, for each stimulus was recalculated based on the subject's prior answers using a Bayesian thresholding method, the Psi-Marginal Adaptive Technique accounting for nuisance parameters like guessing and finger entry errors.^17^ The next stimulus contrast value increased or decreased based upon priors (misses and correct answers) with increasingly smaller contrast steps to refine the final LogCS threshold minimizing the Standard Error. The final CCT value was derived by the Psi-marginal calculation. A summary of the individual responses as LogCS values are reported. The determined threshold also includes a unitless Rabin performance score for clinician convenience that is consistent with the tested contrasts of the original RCCT test in three ranges: less than 90 is normal, 75 to 90 indicates possible CVD, more than 75 indicates CVD. However, a key difference between the RCCT and CCT HD is that scores of greater than 100 can be obtained using CCT HD, whereas 100 is the highest sensitivity score reported using RCCT. The LCD display was routinely calibrated using a USB colorimeter according to manufacturer's specifications. CCT was performed with the patients’ best-corrected visual acuity under photopic and monocular conditions at a distance of 2 feet.

### Statistical Analysis

Three separate analyses evaluating the effects of cataracts on color vision were performed. First, a generalized linear regression model was fit to compare the effect of each filter condition to unfiltered state for the S-cone, M-cone, and L-cone. Generalized estimating equations were used to account for repeated measures under each condition. For the second analysis, a linear regression model fit to evaluate CCT scores for all three cone types on age and lens status was performed. As above, generalized estimating equations were used to account for repeated measures since each individual received binocular testing. The third analysis explored the effects of cataract surgery on color vision using paired *t*-tests to assess changes in CCT scores before and after cataract extraction. All analyses were conducted using R version 1.2.^18^ Bonferroni correction was applied globally to all analyses to account for multiple comparisons using an α of 0.05/18 = 0.0027 threshold for significance. All error bars calculated represent 95% confidence intervals (CIs) with Bonferroni correction.

## Results

### Optimal CCT Test Conditions and Test Validation

**Figure 1.**
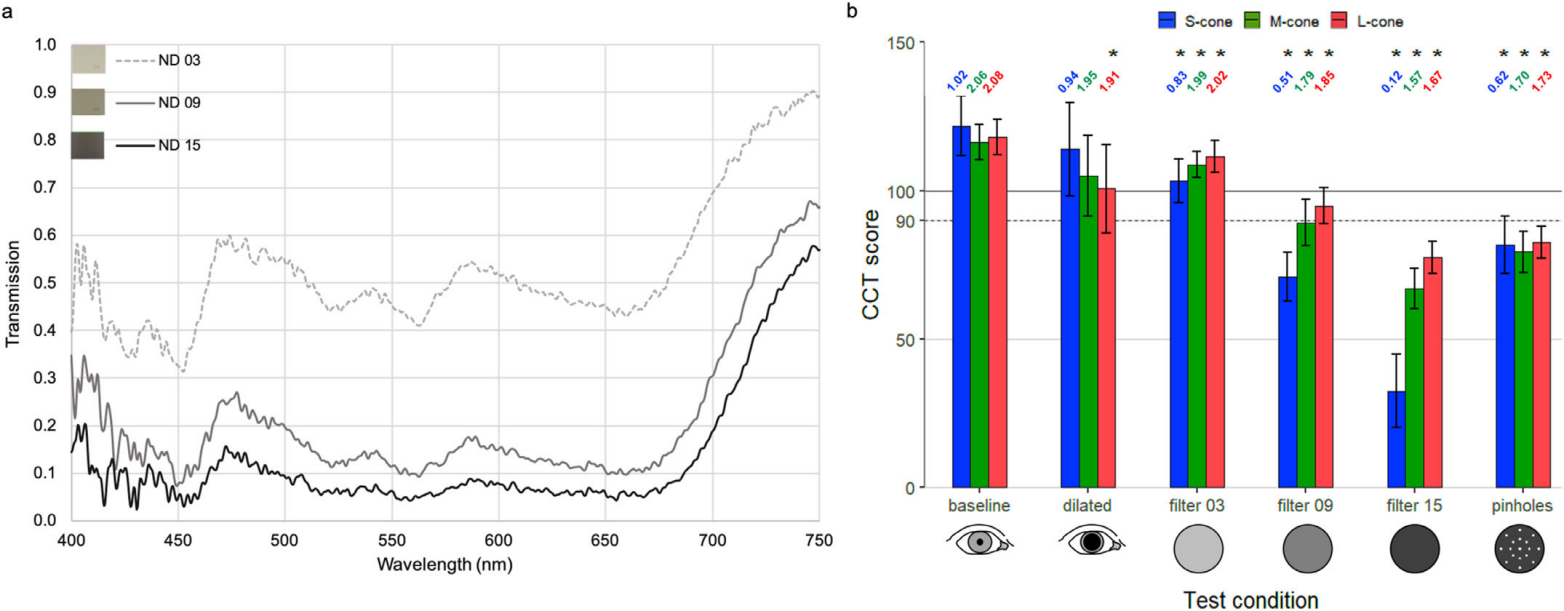
(a) Spectral transmission of NDs. (b) Effect of testing conditions on CCT scores. This was estimated via linear regression with generalized estimating equations to account for repeated measurements (*n* = 18). Error bars represent 95% CI with Bonferroni correction (α = 0.05/18 = 0.0028) and correspond to 2.77 times the standard error of the mean CCT score for each group. *Statistically significant differences from baseline. LogCS scores are included above each bar of Figure 1b.

Figure 1a plots the spectral transmission for each ND filter. Filter 03 permits intermediate visible spectrum transmission. Filters 09 and 15 strongly attenuate the entire visible spectrum. All three filters permit greater transmission in the infrared portion of the spectrum.

Figure 1b plots CCT scores assessed with mydriasis, pinhole correction, and ND filters. Greater CCT scores with the least test variability were observed for the nonmydriatic state without pinholes. The greatest standard deviation for all three cones was observed in the mydriatic state with a significant reduction in L-cone sensitivity. Standard deviation for S-cones, M-cones, and L-cones with mydriasis were 31.6, 24.7, and 28.0, respectively. The CCT scores gradually decreased from baseline when a progressively more dense ND was placed between the CCT and viewer's eye. S-cone class demonstrated the steepest decreases in CCT scores with NDs, whereas the decreases in CCT scores for L- and M-cones were notably less pronounced. S-cone sensitivity decreased from baseline by 15%, 42%, and 73% when tested using ND 0.3, ND 0.9, and ND 1.5, respectively. M-cone sensitivity decreased from baseline by 7%, 24%, and 42% when tested using ND 0.3, ND 0.9, and ND 1.5, respectively. L-cone sensitivity decreased from baseline by 6%, 20%, and 34% when tested using ND 0.3, ND 0.9, and ND 1.5, respectively. All differences in CCT scores from baseline were statistically significant, except for the S-cone and M-cone in the dilated test condition (Fig. 1b).

### Simulated Cataractous Media Opacity (Brown Filters)

**Figure 2.**
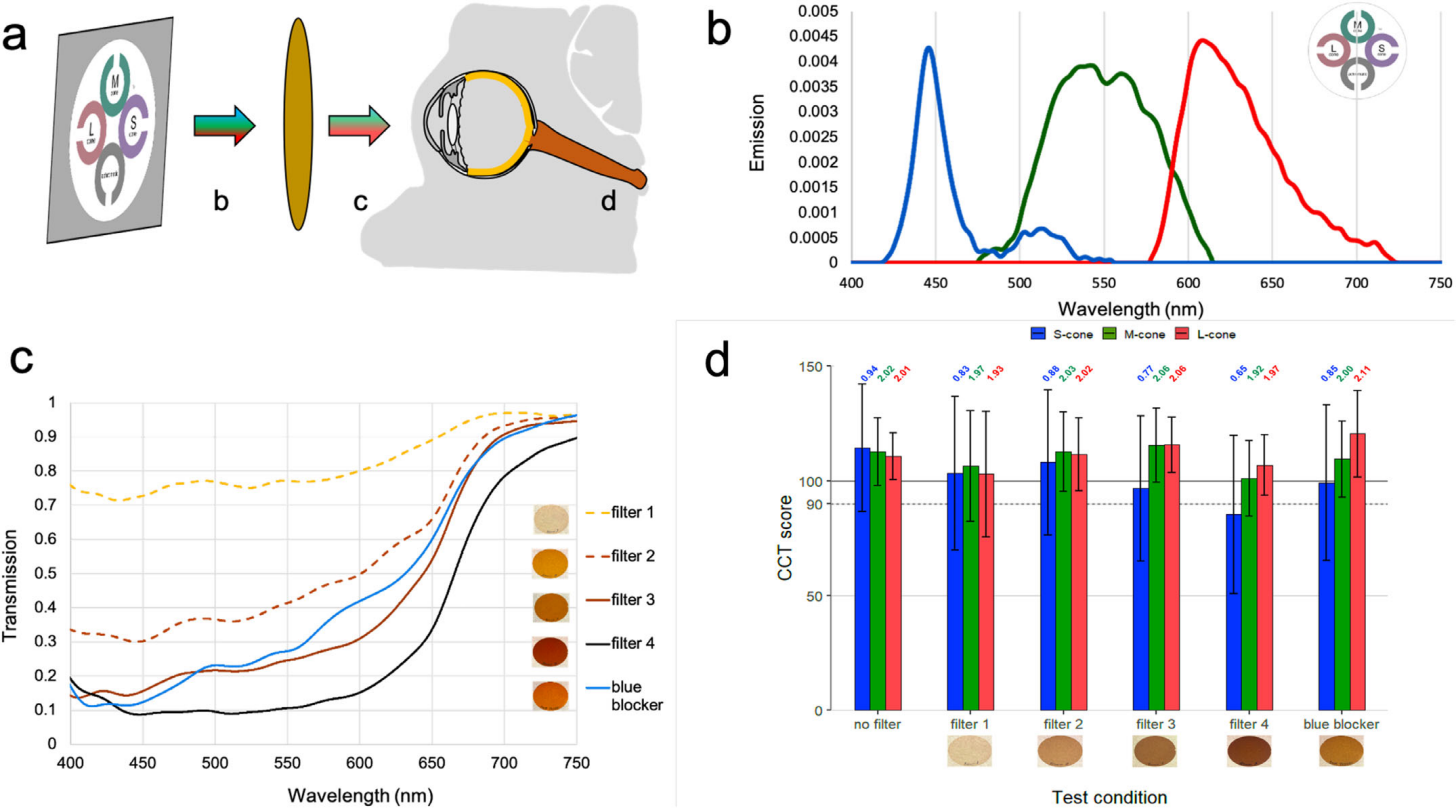
(a) Configuration for measuring cone contrast sensitivity from the ColorDx monitor, with (b) spectral emission of the CCT monitor, (c) spectral transmission of brown filters, and (d) effect of simulated cataractous changes on CCT scores. Error bars represent 95% CI with Bonferroni correction (α = 0.05/18 = 0.0028) and correspond to 2.77 time the standard error of the mean CCT scores for each group. *Statistically significant differences from baseline (*n* = 6). LogCS scores are included above each bar of Figure 2d.

Six phakic eyes of 6 subjects between the ages of 23 and 29 years (25.3 ± 2.4 years) without any ocular history performed CCT using brown filters simulating cataractous media opacity. Figure 2a depicts the testing system for measuring cone contrast sensitivity using different transmission filters. Figure 2b plots the measured emission of the CCT display primaries. The spectral emission ranges for red, green, and blue display primaries are: 577 to 724 nm (peak 608 nm), 475 to 615 nm (peak 541 nm), and 418 to 556 nm (peak 445 nm), respectively.

Figure 2c plots the transmission spectrum for each filter. Filter 1 minimally attenuates shorter wavelengths in the UV-blue spectrum and permits the greatest transmission of light in the visible spectrum of all filters tested. Filter 2, filter 3, and the blue blocker filter allow intermediate visible spectrum transmission. Filter 4 strongly attenuates the entire visible spectrum. Blue blocker transmission surpasses filter 3 transmission at wavelengths of greater than 487 nm. The five filters permit near maximal transmission in the infrared portion of the spectrum.

Figure 2d indicates the subjective response of a test subject observing CCT through brown filters that simulate cataracts with increasing severity. When progressively dense brown filters were placed in front of the test subject's eye, a decrease in CCT scores was seen for the S-cone class. Blue blocker produced a nominal reduction in S-cone sensitivity, whereas the L- and M-cones were nominally unaffected with larger measured CIs. Statistical significance was not achieved for any of the filter conditions when compared with baseline.

### Age-Related Decrease in Color Vision

**Figure 3.**
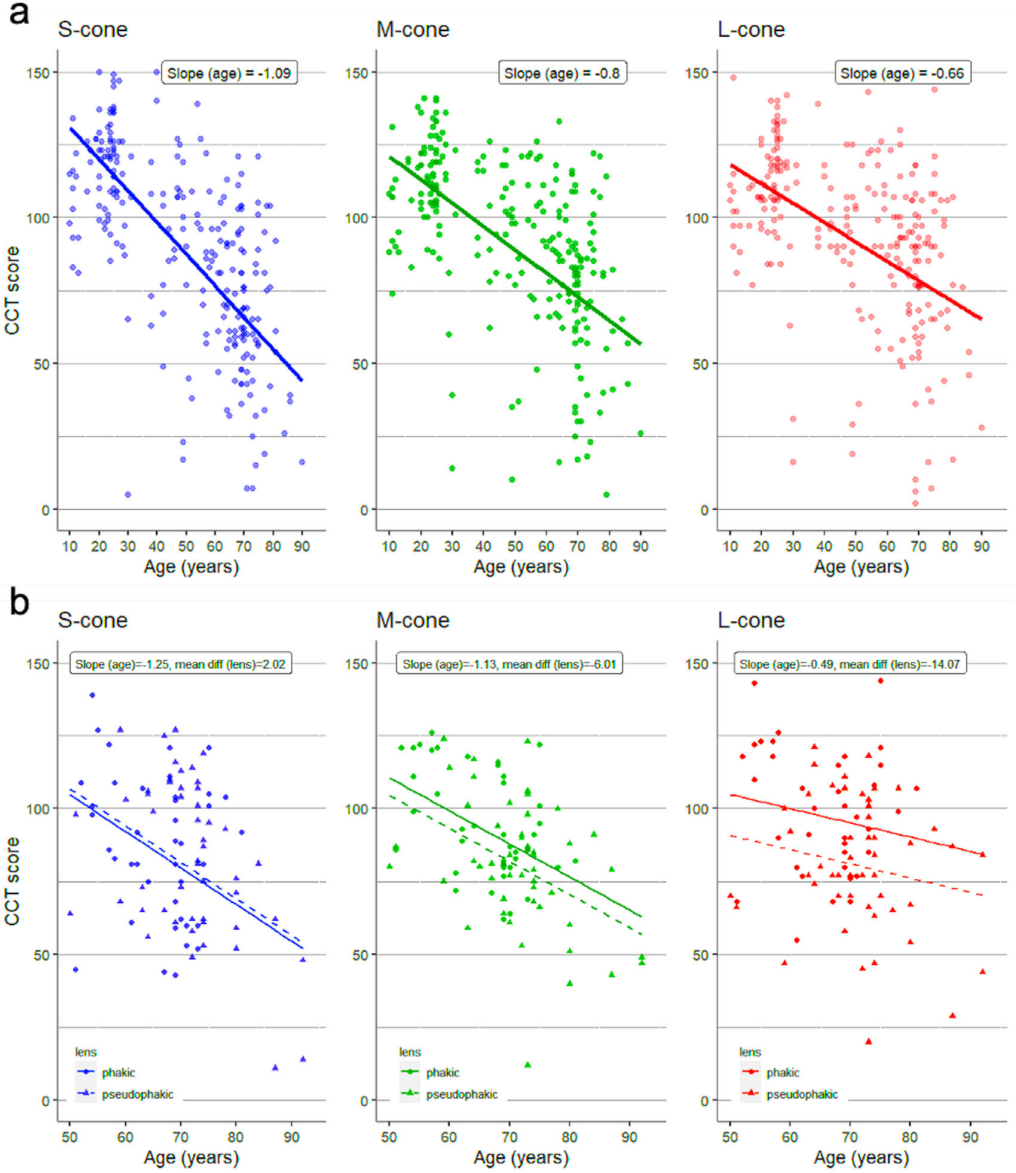
Effect of Age on Color Vision in (a) phakic eyes from 6 years to 90 years old (*n* = 256 eyes) (b) pseudophakic (*n* = 57) eyes vs phakic eyes (*n* = 42) in patients older than 50 years of age.

Phakic eyes (*n* = 256) from subjects between the ages of 6 and 90 years were included in this retrospective study based on having completed CCT performance during their clinical care. Figure 3a summarizes the effect of age on color vision in phakic eyes. CCT scores decreased for all three cone classes with increasing age. The rate of decline was greatest for the S-cones: slope of −1.09 (95% CI, −1.31 to 0.86), and least for the L-cones: slope of −0.66 (95% CI, −0.88 to −0.44).

Figure 3b compares the effect of age on color vision between 57 pseudophakic and 42 phakic eyes in subjects 50 to 94 years old. CCT scores were lower in pseudophakic eyes for the M-cones and L-cones. The rate of decline for the M-cone was: slope of −1.13 (95% CI, −15.31 to 3.29), and least for the L- cones: slope of −0.49 (95% CI, −24.34 to −3.79). CCT scores were higher in pseudophakic eyes for the S-cones. The rate of decline for the S-cones was: slope of −1.25 (95% CI, −10.41 to 14.46). The differences between pseudophakic and phakic eyes for all cone classes were not statistically significant.

### Effects of Cataract on Color Vision

**Figure 4.**
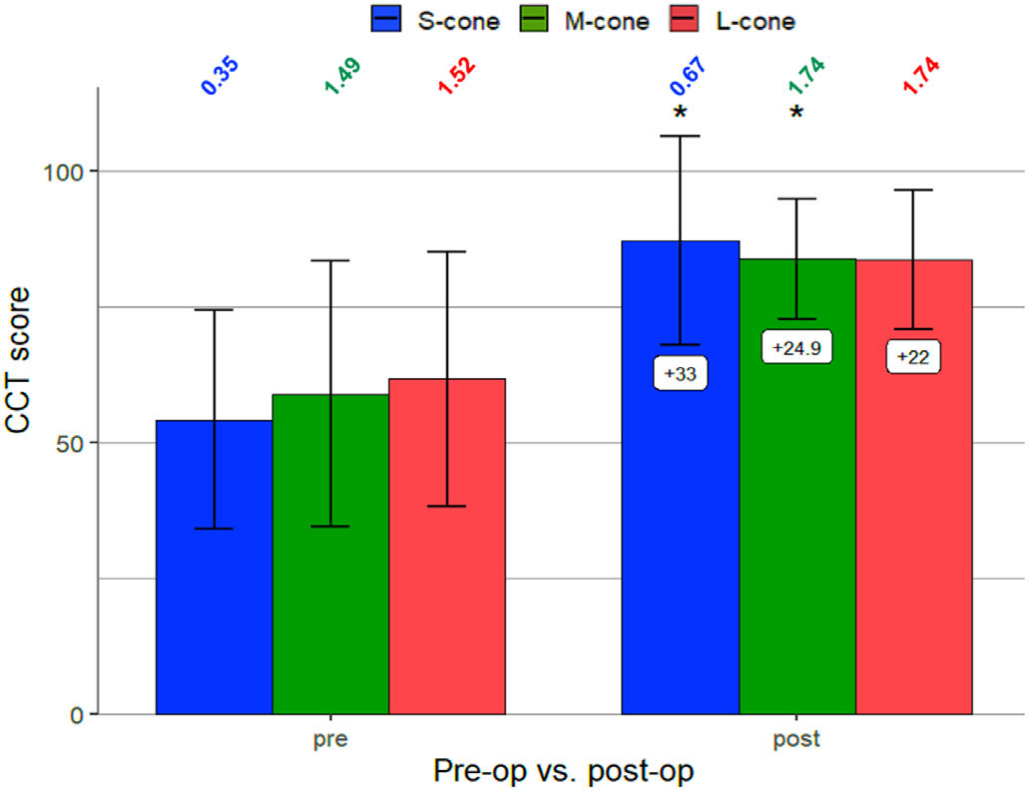
Changes in color vision before and after cataract surgery (*n* = 25). Error bars represent 95% CI with Bonferroni correction (α = 0.05/18 = 0.0028) and correspond to 2.77 times the standard error of the mean CCT score. *Mean changes significant. LogCS scores are included above each bar of Figure 4.

Thirty eyes from 25 subjects between the ages of 51 and 84 years (68.9 ± 6.9 years) were recruited to prospectively study changes in color vision with cataract surgery. Changes in CCT scores before and after cataract extraction are presented in Figure 4. Mean changes between preoperative and postoperative CCT scores for the S-cone, M-cone, and L-cone were 33.0 (95% CI, 8.6 to 57.4), 24.9 (95% CI, 3.8 to 46.0), and 22.0 (95% CI, −3.2 to 47.3), respectively. Changes were only significant for the S-cone and M-cone.

## Discussion

CCT is a modern quantitative color vision and contrast assay that takes approximately 7 minutes for two monocular assessments and is practical for use in clinical evaluation. It has the capability of detecting early stages of systemic, neurologic, and ocular diseases,^15^ including glaucoma^19^ and age-related macular degeneration.^20^ Three separate analyses using CCT were performed to examine the effect of age and lens status on color and contrast vision in three separate scenarios: young people with simulated cataracts, populations of phakic or pseudophakic eyes seen in the course of clinical care, and subjects undergoing cataract surgery.

CCT scores significantly changed depending on testing conditions. Decreases in cone isolation under mydriasis produced the greatest variability in CCT results, whereas decreases in retinal illuminance using pinhole optics may explain the decreased CCT values. Both mydriasis and pinhole optics increase optical aberration which may necessitate CCT testing without mydriasis and without pinhole correction (Fig. 1b). CCT scores decreased in accordance with increasing neutral filter opacity, highlighting the inverse relationship between cone contrast thresholds and lens opacity. Of note, NDs (Fig. 1a) produced uniform attenuation of wavelengths across the visible spectrum and should therefore not alter perceived color. However, S-cone CCT scores demonstrated greater reduction than L- and M-cone classes, indicating that the perceived color of an LCD display is unexpectedly changed by NDs. This result might be explained by the central 2° of the fovea having few to no S-cones.^21^ This effect was minimally reproduced using the cataract-simulating brown filters, which absorb shorter wavelengths more than longer wavelengths (Fig. 2). This outcome may result from greater translucency of the darkest brown filter than the darkest NDs.

CCT scores for all cone classes decreased with advancing age in phakic subjects, as previously found by Fujikawa et al.^22^ S-cone exhibited the greatest rate of decline in sensitivity, highlighting the effect of lenticular aging attenuating shorter wavelengths. Alternatively, age-related decreases in color sensitivity could be attributed to increases in intraocular light scattering independent of wavelength,^23^^,^^24^ decreased cone sensitivity, misalignment of cones from photoreceptor loss, loss of nuclei in the outer nuclear layer, decreases in retinal ganglion cells, or other neurologic factors.^2^^,^^4^^,^^25^

Prior studies comparing color sensitivity between pseudophakic and phakic eyes found either diminished cone contrast sensitivity for the S-cones^26^ or for all three cone classes^2^^,^^27^ in pseudophakic subjects. However, our findings indicate greater S-cone sensitivity in pseudophakic subjects compared with healthy controls, similar to studies performed by Mantyjarvi et al.^28^ This finding could be explained by increased filtering and decreased transmission of blue light in phakic lenses. Subclinical cystoid macular edema, alternatively, could result in retinal tissue breakdown and loss of visual function.^27^ However, none of the patients in our study developed pseudophakic macular edema.

In patients who underwent cataract surgery, cataracts were noted to decrease color sensitivity for all three cone classes to values below normal, with the greatest decrease seen for S-cone class. After cataract surgery, color vision increased to normal levels with the blue cone showing the greatest improvement. This finding is similar to previous studies using quantitatively discrete and continuous assays.^1^^,^^2^ There is some ambiguity as to whether yellow IOLs cause noticeable tritan deficits. Although earlier versions of blue-blocking IOLs have been shown to induce tritan color defects,^29^ other studies have shown no significant difference in color vision under photopic and scotopic light conditions between blue light-filtering IOLs and UV light filtering IOLs.^30^^,^^31^ Notably, the visible density of yellow-tinted IOLs is less remarkable than the lightest tinted brown filter studied here. To our knowledge, this study is the first quantitative demonstration of this phenomenon using a standard clinical visual function test device while simulating cataractous media opacity, population-based evaluation, and perioperative assessment.

A limitation in our study is small sample size for studying progressively dense brown filter lenses. However, test subjects were healthy and reliable test takers. Another limitation is that specific time periods between filter assays were not explicitly controlled. Subjects were tested on one filter, and the subsequent filter was used 30 seconds after normal binocular vision in photopic conditions. This factor may have a small effect by allowing gradual dark adaptation as filters became progressively darker. However, subjects were in photopic conditions during the test and between tests with the total testing time being well below 30 minutes, so dark adaptation would likely be negligible. Additionally, testing with the ND and brown filters could have been initiated using the lowest luminance (darkest filter) first, allowing for a specified adaptation time to adapt to this luminance before testing. This strategy would minimize learning effects by proceeding from worst case to best case scenario. Because light adaptation is rapid, no adaptation would be required when switching to a lighter filter. Another limitation is that the cohort of cataract surgery patients did not exclude early disease. A history of glaucoma,^2^^,^^19^ age-related macular degeneration,^32^ diabetic retinopathy,^33^^,^^34^ or retinal surgeries could contribute to decreased S-cone sensitivity. To further distinguish the effect of cataract surgery on color vision for specific disease states, additional investigation could recruit disease specific sub-populations. Furthermore, the cohort of cataract surgery patients primarily developed nuclear sclerotic cataracts followed by a combination of nuclear sclerotic, cortical, and/or posterior subcapsular cataracts. The effect of cortical and posterior subcapsular cataracts alone were not assessed but are likely to affect cone contrast sensitivity to different degrees, as previously studied by Fristrom and Lundh.^2^

In conclusion, lenticular senescence and cataract formation diminish color sensitivity for all three cone classes, with the greatest decrease for S-wavelength sensitive cones. Cataract surgery can recover a significant proportion of color and contrast vision. CCT can be used to quantify the effect of cataracts and age impose on vision beyond black and white visual acuity testing. Future investigation may reveal the ways CCT testing might be used to guide recommendations for surgery or optimizing lighting conditions during activities of daily life and mitigate fall risks in the elderly.^35^^–^^37^ The degree to which patients subjectively report a more “colorful” and higher contrast world after cataract surgery can be quantified using CCT testing.

## References

[1] Ao M, Li X, Qiu W, Hou Z, Su J, Wang W The impact of age-related cataracts on colour perception, postoperative recovery and related spectra derived from test of hue perception. *BMC Ophthalmol*. 2019; 19(1): 56. 30786855 10.1186/s12886-019-1057-6 PMC6383292

[2] Fristrom B, Lundh BL Colour contrast sensitivity in cataract and pseudophakia. *Acta Ophthalmol Scand*. 2000; 78(5): 506–511. 11037903 10.1034/j.1600-0420.2000.078005506.x

[3] Paramei GV, Oakley B Variation of color discrimination across the life span. *J Opt Soc Am A Opt Image Sci Vis*. 2014; 31(4): A375–A384. 24695196 10.1364/JOSAA.31.00A375

[4] Skomrock LK, Richardson VE Simulating age-related changes in color vision to assess the ability of older adults to take medication. *Consult Pharm*. 2010; 25(3): 163–170. 20363710 10.4140/TCP.n.2010.163

[5] Gillespie-Gallery H, Konstantakopoulou E, Harlow JA, Barbur JL Capturing age-related changes in functional contrast sensitivity with decreasing light levels in monocular and binocular vision. *Invest Ophthalmol Vis Sci*. 2013; 54(9): 6093–6103. 23920364 10.1167/iovs.13-12119

[6] Nguyen-Tri D, Overbury O, Faubert J The role of lenticular senescence in age-related color vision changes. *Invest Ophthalmol Vis Sci*. 2003; 44(8): 3698–3704. 12882826 10.1167/iovs.02-1191

[7] Schneck ME, Haegerstrom-Portnoy G, Lott LA, Brabyn JA Comparison of panel D-15 tests in a large older population. *Optom Vis Sci*. 2014; 91(3): 284–290. 24535417 10.1097/OPX.0000000000000152 PMC4014780

[8] Suzuki TA, Qiang Y, Sakuragawa S, Tamura H, Okajima K Age-related changes of reaction time and p300 for low-contrast color stimuli: Effects of yellowing of the aging human lens. *J Physiol Anthropol*. 2006; 25(2): 179–187. 16679715 10.2114/jpa2.25.179

[9] Fanlo Zarazaga A, Gutierrez Vasquez J, Pueyo Royo V Review of the main colour vision clinical assessment tests. *Arch Soc Esp Oftalmol*. 2019; 94(1): 25–32. 30361001 10.1016/j.oftal.2018.08.006

[10] Birch J Use of the Farnsworth-Munsell 100-Hue test in the examination of congenital colour vision defects. *Ophthalmic Physiol Opt*. 1989; 9(2): 156–162. 2622650 10.1111/j.1475-1313.1989.tb00836.x

[11] Ghose S, Parmar T, Dada T, Vanathi M, Sharma S A new computer-based Farnsworth Munsell 100-hue test for evaluation of color vision. *Int Ophthalmol*. 2014; 34(4): 747–751. 24097078 10.1007/s10792-013-9865-9

[12] Cranwell MB, Pearce B, Loveridge C, Hurlbert AC Performance on the Farnsworth-Munsell 100-Hue test is significantly related to nonverbal IQ. *Invest Ophthalmol Vis Sci*. 2015; 56(5): 3171–3178. 26024100 10.1167/iovs.14-16094

[13] Rabin J, Gooch J, Ivan D Rapid quantification of color vision: the cone contrast test. *Invest Ophthalmol Vis Sci*. 2011; 52(2): 816–820. 21051721 10.1167/iovs.10-6283

[14] Rabin J Cone-specific measures of human color vision. *Invest Ophthalmol Vis Sci*. 1996; 37(13): 2771–2774. 8977494

[15] Rabin J Quantification of color vision with cone contrast sensitivity. *Vis Neurosci*. 2004; 21(3): 483–485. 15518234 10.1017/s0952523804213128

[16] Fraunfelder F, Fraunfelder F, Chambers W *Clinical Ocular Toxicology*. 1 ed. Saunders Elsevier; 2008.

[17] Prins N The psi-marginal adaptive method: How to give nuisance parameters the attention they deserve (no more, no less). *J Vis*. 2013; 13(7): 3. 10.1167/13.7.3 23750016

[18] R Core Team. *R: A language and environment for statistical computing*. Vienna, Austria: R Foundation for Statistical Computing URL http://www.R-project.org/. 2019.

[19] Niwa Y, Muraki S, Naito F, Minamikawa T, Ohji M Evaluation of acquired color vision deficiency in glaucoma using the Rabin cone contrast test. *Invest Ophthalmol Vis Sci*. 2014; 55(10): 6686–6690. 25168899 10.1167/iovs.14-14079

[20] Cocce KJ, Stinnett SS, Luhmann UFO, et al. Visual Function Metrics in Early and Intermediate Dry Age-related Macular Degeneration for Use as Clinical Trial Endpoints. *Am J Ophthalmol*. 2018; 189: 127–138. 29477964 10.1016/j.ajo.2018.02.012 PMC6043161

[21] Curcio CA, Hendrickson AE Organization and development of the primate photoreceptor mosaic. *Progress in Retinal Research*. 1991; 10: 89–120.

[22] Fujikawa MS, Muraki YN, Ohji M. Evaluation of clinical validity of the Rabin cone contrast test in normal phakic or pseudophakic eyes and severely dichromatic eyes. *Acta Ophthalmol,* 2018; 96(2): e164–e167. 28556475 10.1111/aos.13495 PMC5836892

[23] Fiorentini A, Porciatti V, Morrone MC, Burr DC Visual ageing: unspecific decline of the responses to luminance and colour. *Vision Res*. 1996; 36(21): 3557–3566. 8977022 10.1016/0042-6989(96)00032-6

[24] Michael R, Bron AJ The ageing lens and cataract: a model of normal and pathological ageing. *Philos Trans R Soc Lond B Biol Sci*. 2011; 366(1568): 1278–1292. 21402586 10.1098/rstb.2010.0300 PMC3061107

[25] Simunovic MP Acquired color vision deficiency. *Surv Ophthalmol*. 2016; 61(2): 132–155. 26656928 10.1016/j.survophthal.2015.11.004

[26] Jay JL, Gautam VB, Allan D Colour perception in pseudophakia. *Br J Ophthalmol*. 1982; 66(10): 658–662. 6981423 10.1136/bjo.66.10.658 PMC1039892

[27] Knowles PJ, Tregear SJ, Ripley LG, Casswell AG Colour vision in diabetic and normal pseudophakes is worse than expected. *Eye (Lond)*. 1996; 10(Pt 1): 113–116. 8763315 10.1038/eye.1996.19

[28] Mantyjarvi M, Syrjakoski J, Tuppurainen K, Honkonen V Colour vision through intraocular lens. *Acta Ophthalmol Scand*. 1997; 75(2): 166–169. 9197565 10.1111/j.1600-0420.1997.tb00116.x

[29] Rubin RM, Rabin J, Ivan D, et al. The impact of blue-blocking intraocular lenses on S cone color vision. *Investigative Ophthalmology & Visual Science*. 2007; 48(13): 6011–6011.

[30] Zhu XF, Zou HD, Yu YF, Sun Q, Zhao NQ Comparison of blue light-filtering IOLs and UV light-filtering IOLs for cataract surgery: a meta-analysis. *PLoS One*. 2012; 7(3): e33013. 22412976 10.1371/journal.pone.0033013 PMC3296774

[31] Greenstein VC, Chiosi F, Baker P, et al. Scotopic sensitivity and color vision with a blue-light-absorbing intraocular lens. *J Cataract Refract Surg*. 2007; 33(4): 667–672. 17397741 10.1016/j.jcrs.2006.12.012 PMC1913934

[32] Arden GB, Wolf JE Colour vision testing as an aid to diagnosis and management of age related maculopathy. *The British Journal of Ophthalmology*. 2004; 88(9): 1180–1185. 15317712 10.1136/bjo.2003.033480 PMC1772298

[33] Cho NC, Poulsen GL, Ver Hoeve JN, Nork TM Selective loss of S-cones in diabetic retinopathy. *Arch Ophthalmol*. 2000; 118(10): 1393–1400. 11030822 10.1001/archopht.118.10.1393

[34] Fristrom B Peripheral and central colour contrast sensitivity in diabetes. *Acta Ophthalmol Scand*. 1998; 76(5): 541–545. 9826036 10.1034/j.1600-0420.1998.760506.x

[35] Ivers RQ, Cumming RG, Mitchell P, Attebo K Visual impairment and falls in older adults: the Blue Mountains Eye Study. *J Am Geriatr Soc*. 1998; 46(1): 58–64. 9434666 10.1111/j.1532-5415.1998.tb01014.x

[36] McCarty CA, Fu CL, Taylor HR Predictors of falls in the Melbourne visual impairment project. *Aust N Z J Public Health*. 2002; 26(2): 116–119. 12054328 10.1111/j.1467-842x.2002.tb00902.x

[37] Saftari LN, Kwon O-S Ageing vision and falls: a review. *Journal of Physiological Anthropology*. 2018; 37(1): 11–11. 29685171 10.1186/s40101-018-0170-1 PMC5913798

